# Vibrotactile Discrimination Training Affects Brain Connectivity in Profoundly Deaf Individuals

**DOI:** 10.3389/fnhum.2017.00028

**Published:** 2017-02-06

**Authors:** Andrés A. González-Garrido, Vanessa D. Ruiz-Stovel, Fabiola R. Gómez-Velázquez, Hugo Vélez-Pérez, Rebeca Romo-Vázquez, Ricardo A. Salido-Ruiz, Aurora Espinoza-Valdez, Luis R. Campos

**Affiliations:** ^1^Instituto de Neurociencias, Universidad de GuadalajaraGuadalajara, Mexico; ^2^Organismo Público Descentralizado Hospital Civil de GuadalajaraGuadalajara, Mexico; ^3^Departamento de Ciencias Computacionales, Centro Universitario de Ciencias Exactas e Ingenierías, Universidad de GuadalajaraGuadalajara, Mexico; ^4^Facultad de Informática, Ciencias de la Comunicación y Técnicas Especiales, Universidad de MorónBuenos Aires, Argentina

**Keywords:** brain development, event-related potentials, sensory systems, brain connectivity, deafness/hearing loss, vibrotactile stimulation, learning and plasticity

## Abstract

Early auditory deprivation has serious neurodevelopmental and cognitive repercussions largely derived from impoverished and delayed language acquisition. These conditions may be associated with early changes in brain connectivity. Vibrotactile stimulation is a sensory substitution method that allows perception and discrimination of sound, and even speech. To clarify the efficacy of this approach, a vibrotactile oddball task with 700 and 900 Hz pure-tones as stimuli [counterbalanced as target (T: 20% of the total) and non-target (NT: 80%)] with simultaneous EEG recording was performed by 14 profoundly deaf and 14 normal-hearing (NH) subjects, before and after a short training period (five 1-h sessions; in 2.5–3 weeks). A small device worn on the right index finger delivered sound-wave stimuli. The training included discrimination of pure tone frequency and duration, and more complex natural sounds. A significant P300 amplitude increase and behavioral improvement was observed in both deaf and normal subjects, with no between group differences. However, a P3 with larger scalp distribution over parietal cortical areas and lateralized to the right was observed in the profoundly deaf. A graph theory analysis showed that brief training significantly increased fronto-central brain connectivity in deaf subjects, but not in NH subjects. Together, ERP tools and graph methods depicted the different functional brain dynamic in deaf and NH individuals, underlying the temporary engagement of the cognitive resources demanded by the task. Our findings showed that the index-fingertip somatosensory mechanoreceptors can discriminate sounds. Further studies are necessary to clarify brain connectivity dynamics associated with the performance of vibrotactile language-related discrimination tasks and the effect of lengthier training programs.

## Introduction

The rationale that auditory deprivation could benefit sensory modalities that remain intact (Mayberry, 2002; Auer et al., 2007) underlies the exploration of vibrotactile stimulation as an alternative sound perception method for the population with profound bilateral deafness by enabling discrimination of sound and even spoken language. Several studies have explored speech perception via the somatosensory system employing vibrotactile stimulation devices (Rothenberg and Molitor, 1979; Plant and Risberg, 1983; Bernstein et al., 1998; Auer et al., 2007). The search for alternate communication methods is necessary primarily because early oral language acquisition is a challenge for profoundly deaf individuals and has important implications for neurodevelopment and, consequently, cognition (Youniss et al., 1971; Peterson and Siegal, 1995; Mayberry, 2002; Marschark and Hauser, 2008). Hence, implementing novel technologies and training programs that facilitate vibrotactile perception and discrimination of sounds within the language spectrum could well have a positive impact on oral language development.

In recent decades, the effects of early auditory deprivation on brain organization due to neuroplasticity in developmental stages have been explored, primarily in the sensory cortices (Huttenlocher, 2002) and language-related areas (Huttenlocher and Dabholkar, 1997; Neville and Bavelier, 1998). The recruitment of auditory cortices for processing sign language (Chlubnová et al., 2005), visual tasks (Finney et al., 2003), and vibrotactile stimulation (Levänen et al., 1998; Auer et al., 2007), are cross-modal changes related to profound deafness (Merabet and Pascual-Leone, 2010).

Advances in instrumentation technology for sensory substitution have opened up new opportunities to develop practical and inexpensive systems to compensate for sensory loss (Bach-y-Rita and Kercel, 2003). Sensory substitution, studied by Bach-y-Rita since 1969, had the primary goal of providing users with environmental information through a human sensory channel different from the one normally used. Many studies have demonstrated that this phenomenon can occur across sensory systems, such as touch-to-sight (Bach-y-Rita, 2004), and that visual, auditory and modified tactile information can be processed by skin mechanoreceptors to achieve tactile-vision substitution and tactile-auditory substitution (Kaczmarek et al., 1991). Early studies had used vibrotactile stimulation devices to evaluate somatosensory perception of speech in profoundly deaf and normal-hearing (NH) individuals (Risberg and Lubker, 1978; Rothenberg and Molitor, 1979). They found that lip-reading and the perception of prosodic elements of language were facilitated, and that these abilities improved greatly with training. These results were replicated by Reed (1996) in deaf-blind individuals using the TADOMA method that also relies on vibrotactile perception.

Vibrotactile stimulation produces a characteristic cortical response that is distinguishable and, therefore, easily evaluated. Using magnetoencephalography (MEG), Caetano and Jousmäki (2006) demonstrated the convergence of vibrotactile input on the superior temporal cortex of normal-hearing adults, as had been reported previously in a congenitally deaf adult (Levänen et al., 1998). Recently, a vibrotactile-related endogenous neural response was mapped for purposes of surgical resection (Wahnoun et al., 2015). Hegner et al. (2010) found that different neuronal mechanisms underlie tactile and vibrotactile cortical processing, in which cortical representations vary depending on the nature of the stimuli. Their results also suggest that the right hemisphere is more dominant in pattern than frequency vibrotactile discrimination, which could be attributed to the differences between spatial (pattern) and temporal (frequency) processing discrimination. Furthermore, Ammirante et al. (2013) found that deaf individuals can discriminate between same-sex talkers based on vibrotactile stimulation alone. Their findings suggest that the discrimination of complex vibrotactile stimuli involves cortical integration of spectral information filtered through frequency-tuned skin mechanoreceptors.

The study of electrical brain activity depicts the characteristics of neural changes across time, along with the connectivity that supports those changes. In this context, the aim of the present study was to explore underlying learning-related electrophysiological changes in subjects with profound deafness and normal-hearing controls after a short training period in vibrotactile sound discrimination by applying two EEG analysis techniques: event-related potentials (ERPs) and graph theory tools.

Event-related brain potentials have been utilized to study time-locked cerebral processes while performing behavioral tasks that involve attention and working memory resources. Specifically, the P300 component has been extensively studied as an index of updating memory representations (Donchin and Coles, 1988; Polich, 2007) and general cognitive performance used to monitor illness evolution in clinical models (Sumiyoshi et al., 2006; Madan et al., 2007; Kalita et al., 2009; see Duncan et al., 2009 for a review; Lori et al., 2011). In the 1980s, Neville and colleagues published the first electrophysiological studies of the cortical distribution of visual-evoked potentials in deaf individuals, proving that primary sensory cortices can assume other functions in the absence of input in one sensory modality, and that cortical representation and connectivity are determined by the input received during early developmental stages (Neville et al., 1983; Neville and Lawson, 1987). Similar cross-modal plasticity has been observed in early-blind individuals in which the visual cortices are sensitive to attentional changes in the auditory environment (Kujala et al., 2005). However, few studies have approached the evaluation of ERP responses while perceiving vibrotactile stimulation.

Graph theory-based analysis has been widely used to study models of neural networks, anatomical and functional connectivity based on fMRI (Salvador et al., 2005; Achard et al., 2006; Astolfi et al., 2007), MEG (Stam, 2004; Van Wijk et al., 2010), and EEG (Sporns et al., 2000; Astolfi et al., 2006; Espinoza-Valdez et al., 2016). Bernhardt et al. (2015) define graph theory as a mathematical framework to quantify topological properties of complex interconnected systems. This has been applied to study the topological properties of networks –i.e., sets of nodes on which edges are defined– derived from brain imaging and electrophysiological data. A ‘graph’ refers to an abstract representation of a network, in which nodes represent brain regions and edges represent connections. Several graph measures make it possible to characterize graph topologies in terms of efficiency transfer and the balance between “segregation” and “integration” (see Bullmore and Sporns, 2012, for an extensive review).

Graph theory-based analysis provides a method for quantifying brain networks using a reduced number of meaningful biological measures that are easily determined. Thus, it is argued that graph metrics characterize brain networks (Fraga González et al., 2016). In the context of ERPs, calculating partial directed coherence (PDC) based on such time-variant multivariate autoregressive models and measures as centrality or modularity have emerged as useful tools for assessing connectivity between different brain locations (Schelter et al., 2006; Keller et al., 2014; Rodrigues and Baccalá, 2015). Recently, graph theory-based methods have been used to explore the cortical reorganization of functional networks in prelingual deaf adolescents (Li et al., 2016). Indeed, ERPs and graph analyses are complementary, not mutually exclusive techniques (eg., Mutlu et al., 2012) that can contribute to a better understanding of how profoundly deaf individuals learn to discriminate sound using a novel sensory pathway.

Several studies using fMRI have revealed an overlap between attention and working memory networks over visual, parietal and frontal areas (Gazzaley et al., 2007; Mayer et al., 2007; Gazzaley and Nobre, 2012), findings which support the view that these cognitive functions share neural resources and are governed by the fronto-parietal attention network (Corbetta et al., 2002; Zanto et al., 2011). In fact, these fronto-parietal regions have been identified as containing a task-positive network (Fox et al., 2005) or “fronto-parietal control system” (Vincent et al., 2008) that, in terms of connectivity, has been described as a “flexible hub” that adjusts its connectivity patterns to task requirements (Cole et al., 2013). Interestingly, Jackson et al. (2016) have found that the fronto-parietal cortex adjusts its representation of visual objects, suggesting that this effect is not stimulus-modality specific.

In summary, a powerful, early link between human speech and cognition guides infant development and casts a wide facilitative net for the fundamental cognitive capacities that underlie other core learning processes (Vouloumanos and Waxman, 2014; Ferguson and Lew-Williams, 2016; Ferguson and Waxman, 2016). There is broad evidence in the literature to support the notion that neurodevelopmental deficits in profound deafness affect cognitive flexibility (Courtin, 1997; Kushalnagar et al., 2010). Moreover, dynamic changes in fronto-parietal connectivity have been associated with cognitive flexibility. Nevertheless, information on how underlying neural processes adjust when deaf individuals are trained to vibrotactile discriminate sounds is still scanty.

Our experiment aimed to explore, comparatively, how training in vibrotactile sound discrimination affects electrophysiological processing and functional connectivity in profoundly deaf and NH individuals. Due to their early sensory deprivation and impoverished language acquisition, deaf individuals might obtain fewer benefits from a short training period. Thus, we hypothesized that they will have higher amplitudes in the P300 component –before and after training– compared to NH controls, with less frontal and parietal functional disengagement, as quantified by connectivity metrics. To our knowledge, the present study is the first to use graph theory-based analysis to explore changes in brain connectivity due to training in vibrotactile discrimination of sound within the language frequency spectrum.

## Materials and Methods

### Subjects

Fourteen right-handed subjects with prelingual profound bilateral deafness (seven males; mean: 21.96, *SD* = 6.63 years), and 14 age-and-sex-matched normal-hearing controls (mean: 21.93, *SD* = 5.02 years) volunteered to participate. Most control subjects were family members with similar demographic characteristics. Clinical interviews determined that no participants had personal or family histories of psychiatric, neurological or neurodegenerative illness. All participants also had normal neurological examinations and normal baseline EEGs. All deaf participants were Mexican Sign Language (MSL) users. Thirteen had received proper sign language instruction late in childhood (after age seven), most upon entering primary school. Only one participant was born to deaf parents and had learned MSL at home as his maternal language.

The study was reviewed and approved by the Ethics Committee at the Neuroscience Institute (Universidad de Guadalajara). A professional interpreter translated all forms, questionnaires and instructions into MSL, and all volunteer participants or the parents of under-aged subjects gave their informed written consent.

### Audiological Testing

Using a Maico MA-41 Portable Audiometer with audio over-ear headphones and bone-conduction headphones, hearing threshold measurements were taken at six octaves: 250, 500, 1000, 2000, 4000, and 8000 Hz. Pure-tone air and bone conduction audiometries were performed to confirm profound bilateral sensorineural hearing loss with a pure-tone average (PTA) greater than 90 decibels (dB) in the deaf participants, and normal-hearing levels in the controls.

### Design and Procedure

We studied cerebral electric activity in 14 profoundly deaf and 14 NH participants using a classic oddball paradigm. Since the design was longitudinal, the experimental task was performed twice by the same individuals. An initial baseline EEG recording was made, followed by a second one after five vibrotactile sound discrimination sessions (1-h duration, 2–3 times a week). The sessions focused on training vibrotactile discrimination of frequency and duration properties of sound, and involved exercises with three pure-tone sequences of varying levels of difficulty, as well as the discrimination of a total of 12 complex sounds, such as natural animal and object sounds. See Data Sheet 1 for detailed training program description.

Participants were comfortably seated in a quiet, well-lit room. The vibrotactile oddball paradigm consisted of a train of 150 randomly presented stimuli, with a duration of 200 ms (ISI: 1500 ms) and a 20:80 rare stimulus frequency. The stimuli consisted of 700 and 900 Hz pure-tones; infrequent target and frequent standard conditions were counterbalanced across subjects. Participants were instructed to look at a cross-shaped fixation point on the center of a 19-inch SVGA monitor (refresh rate: 100 Hz) to minimize ocular artifacts, and to respond by pressing the left control key with their left index finger upon target stimulus detection. Sound-wave stimuli were delivered by a portable stimulator system (adapted model of the SEVITAC-D^®^) worn on the right index finger and connected directly to the computer’s audio output (volume level set at 80 dB SPL). Stimuli presentation was controlled by MINDTRACER-2.0 software (Neuronic, S.A.). The portable stimulator system has a sound range frequency of 0–10 kHz and consists of a tiny flexible plastic membrane with a 78.5-mm^2^ surface area that vibrates on the tip of the index finger in response to sound pressure waves via analog transmission.

During task execution, the NH participants wore earplugs and circumaural hearing protection, and were exposed to background white-noise (70 dB SPL). Also, they placed their right hand inside a sound-attenuated box to ensure the stimuli were not auditorily perceptible and were only processed via somatosensory pathways.

### ERP Acquisition

#### Recording

EEG activity was recorded from the Fp1, Fp2, F3, F4, F7, F8, C3, C4, P3, P4, O1, O2, T3, T4, T5, T6, Fz, Cz and Pz scalp electrode sites, following the 10–20 system and using a commercial electro cap. EOGs were recorded from the outer canthus and infraocular orbital ridge of the right eye. All recording sites were referred to linked mastoids. Inter-electrode impedances were below 5 kΩ at 30 Hz. EEG and EOG signals were amplified at a band pass of 0.05–30 Hz (3-dB cutoff points of 6 dB/octave roll-off curves) with a sampling period of 5 ms on the MEDICID-04 system (Neuronic S.A.). Single trial data were examined off-line for averaging and analysis.

#### Behavioral Measures

Correct and incorrect responses were marked automatically on the EEGs by the software; reaction times were recorded simultaneously.

#### Signal Averaging

Stimulus onset was taken as the initial time instant (t0). ERP time windows were obtained from 100 ms before the onset of the stimuli to 1000 ms after it. Fifteen artifact-free trials (50% of the infrequent trials) were averaged for each condition to obtain the P300 components. A pre-stimulus period of 100 ms was used for baseline correction. Epochs of data on all channels were excluded from the averages when the voltage in a given recording epoch exceeded 100 μV on any EEG or EOG channel. Each individual ERP reached a standard deviation rate (SDR) below 1.1 and a residual noise level (RNL) below 2. Epochs with artifacts were also rejected by visual inspection performed by two group-blinded experts.

#### Graph Analysis

Several estimators based on time and EEG frequencies have been developed to evaluate brain connectivity. While the two classic estimators of correlation and coherence generate information on directionality, they do not provide data on causal relationships. Therefore, PDC (π_ij_, where i and j represent two electrodes of the EEG array) was defined for the purpose of estimating the direct flows between channels (Baccalá and Sameshima, 2001).

EEG epochs were pre-processed to reduce noise and artifacts using blind source separation and wavelet de-noising procedures before calculating PDC (Romo-Vázquez et al., 2012). To estimate brain connectivity, PDC was computed from a 1.5-second time window beginning with the appearance of an “infrequent stimuli” selected halfway through EEG recording, simultaneously with performance of the oddball paradigm. This procedure generated a connectivity matrix containing directivity information (see Data Sheet 2), which was binarized using the criterion π_ij_ = 1 if 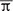
ij > 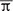
*ij* and π_ij_ = 0, otherwise, where 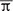 represents the values averaged in the frequency of PDC elements (π; Espinoza-Valdez et al., 2016). However, while PDC does operate in the frequency domain, its coefficients do not have direct correspondence to the power spectrum.

Since PDC is not sensitive to volume conduction and we were interested in exploring not only causality relationships, but also the amount of energy that flowed between the channels, the cross-power spectrum density (CPSD) was also computed for the window under study. The CPSD matrix was averaged, thresholded and binarized. As these two matrixes were multiplied –CPSD × PDC– the resulting matrix contains both the power information from each channel and the notion of causality.

This methodology was applied to each EEG recording in the database, which contained 14 recordings from deaf participants pre-training and 14 post-training, with those of their paired control subjects (56 recordings in total). The matrixes obtained were averaged and constitute the basis of the graphs. An 8 × 8 connectivity matrix was obtained that provides information on the power of the connections between each scalp location with respect to all the others, while ignoring whether the other electrodes were placed on the same –or contralateral– brain hemisphere. The rationale for considering the mean value of connectivity between each electrode and all others across hemispheres is to attempt to measure –via a specific coefficient– the relative relevance of each location in specific regions of interest (ROI). Higher values for these coefficients indicate that one precise location is more important for whole brain connectivity. An additional connectivity analysis was performed to separately evaluate intra- and inter-hemispheric relationships, omitting the midline scalp locations. The resulting matrix was interpreted in graph form.

### Data Analysis

Behavioral data (correct responses, incorrect responses, and reaction times) were analyzed using repeated-measures ANOVAs. The event-related brain potential measures were assessed using Randomized-block ANOVAs [group (2) × condition (2: pre-, post-training) × hemisphere (P3, P4)] with maximum voltage across each time window. The amplitude and latency of each ERP component was quantified at the highest peak within an *a priori* time-window range selected between 270 and 650 ms. Greenhouse-Geisser corrections to the df were applied as needed, with the corrected probabilities reported.

#### Statistical Analysis of Brain Connectivity

According to the topographic distribution of the fronto-parietal network, graph analysis was performed in two different ROIs: (a) fronto-central (Fz, Cz, F3, F4, F7, F8, C3 and C4 scalp locations); and, (b) posterolateral areas (Pz, P3, P4, T3, T4, T5, T6, O1 and O2). The connectivity coefficients for both ROIs were obtained from the EEG epochs (1.5 s) that corresponded to correct responses while detecting the infrequent stimuli. These were analyzed with Welch *t*-tests for two samples (Deaf, NH) in two conditions: pre- and post-training. This analysis was chosen to more accurately represent the functional dynamic of the cognitive processing period, corresponding to the electrophysiological measures portrayed in the ERP waveforms.

In order to evaluate intra-hemispheric connectivity, specifically lateralization effects, two other ROIs were analyzed: (a) left hemisphere (F3, F7, C3, P3, T3, T5 and O1 leads); and, (b) right hemisphere (F4, F8, C4, P4, T4, T6 and O2 leads) by obtaining coefficients from the same EEG time windows.

The mean value of connectivity was calculated for each electrode with respect to all others in each ROI. This connectivity value was estimated by averaging the connectivity matrix across the columns while excluding the diagonal. In this way, mean connectivity values of 8 × 14 and 9 × 14 were obtained for the fronto-central and posterolateral analyses, respectively. In the intra-hemispheric analysis, mean connectivity values of 7 × 14 were obtained for each hemisphere.

## Results

The electrophysiological and behavioral data from two deaf subjects and their matched controls had to be excluded from the ERP analysis because the minimum number of artifact-free windows for ERP averaging was not obtained.

### Behavioral Measures

Significant changes were found in pre- and post-training performance, but no such changes were demonstrated across training conditions between groups. Both groups increased the number of correct responses [*F*_(1,22)_ = 6.604, *p* < 0.05, η = 0.231; mean standard error (MSE) = 1.59] and decreased the number of incorrect responses [*F*_(1,22)_ = 22.232, *p* < 0.001, η = 0.503; MSE = 1.48]. The latter were defined as trials in which individuals failed to discriminate the target from the standard stimuli by responding to the standard. Discrimination task behavioral accuracy rates based on mean correct responses for the profoundly deaf group were 48% before training and 62% after, while those of the control group were 42% before and 59% after. Therefore, after completing only a short training period of five 1-h sessions, all subjects had learned to identify the rare stimuli and were less likely to make detection errors in the vibrotactile sound discrimination oddball paradigm. There were no statistically significant changes in reaction times due to training or between groups.

### ERP Results

**FIGURE 1:**
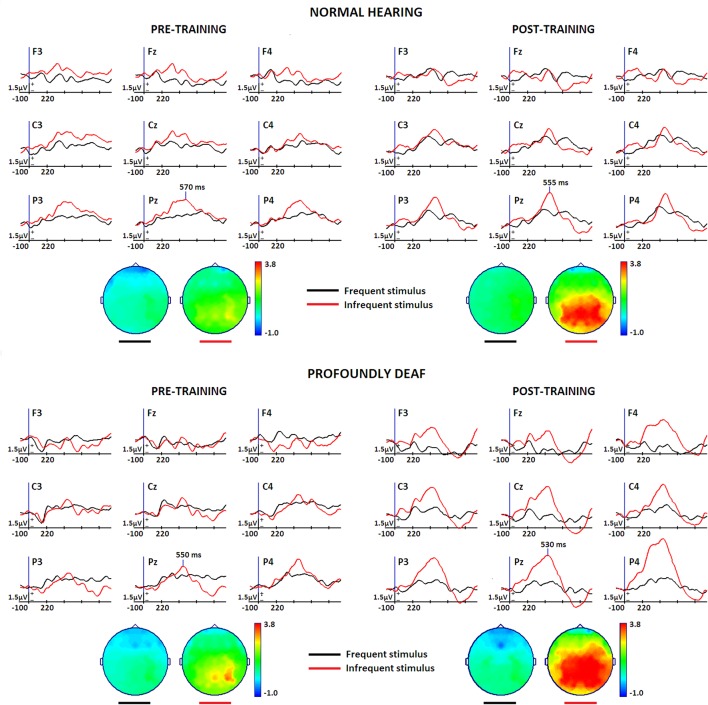
**Midline and vicinity group grand-mean ERP waveforms for the differences between rare target (red) and frequent non-target (black) conditions while performing a vibrotactile tone-frequency discrimination oddball task, before and after a short training period in vibrotactile recognition**. The normal-hearing group (*n* = 12) is represented at the top of the Figure, and deaf participants (*n* = 12) at the bottom. Topographic maps show the voltage distribution at maximum peak latency. A pre-stimulus period of 100 ms was used for baseline correction. Stimulus onset corresponds to the initial time instant in the Figure.

**Figure 1** presents the effects of training on the mean voltage amplitude for midline event-related brain potentials while performing the experimental task in both groups. The topographic maps show the P300 scalp distributions. According to visual inspection, maximum peak latencies were estimated in the grand-averaged waveforms for each group and condition at the Pz electrode. Amplitude data from the parietal electrode locations that showed the most robust changes (P3, P4) in the P300 component were analyzed. The maximum P3 and P4 voltages were identified in each individual mean ERP in both groups and conditions (pre-, post-training) to comparatively evaluate the ERP waveforms while performing the vibrotactile discrimination task.

The results of these analyses indicate a significant increase of the P300 amplitude due to training [*F*_(1,22)_ = 6.078, *p* < 0.05, η = 0.216]. Though between-group differences did not reach statistical significance, a clear tendency for the deaf group to exhibit greater voltage amplitudes is visible in the grand-average waveforms and on the topographical maps. Latency analysis showed no significant effects for training or group differences. However, parietal right hemisphere lateralization during vibrotactile discrimination of sound was more significant in the profoundly deaf group, as demonstrated by a significant group × hemisphere interaction effect [*F*_(1,22)_ = 4.622, *p* < 0.05, η = 0.174].

### Brain Connectivity Differences

**FIGURE 2:**
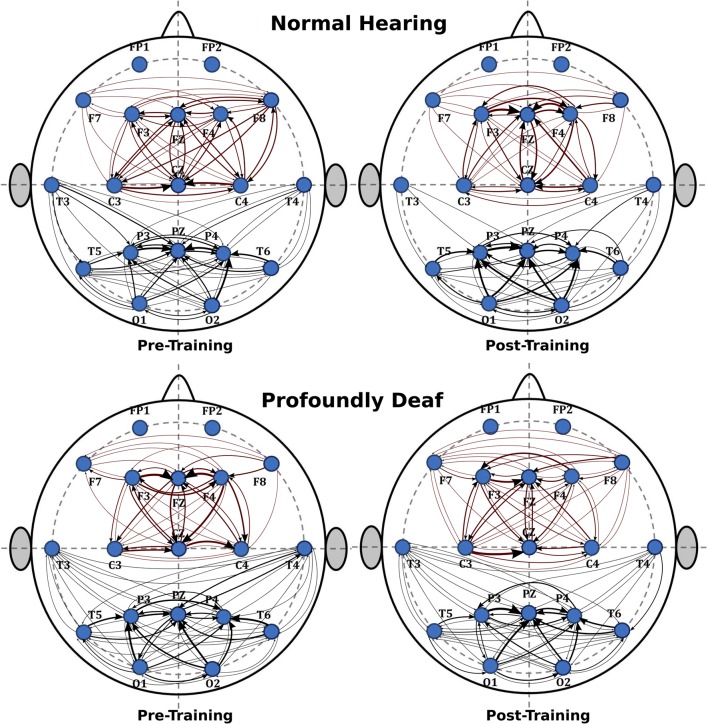
**Sequence of graphs representing brain organization in pre- and post-training conditions in both groups (normal-hearing, profoundly deaf)**. Two brain neural arrangements were studied as main regions of interest: fronto-central and posterolateral areas. The graph structure is defined by the probability of connections between brain regions where the thickness of the lines represents the level of connectivity (CPSD × PDC).

**Figure 2** shows the main changes in brain connectivity associated with training in vibrotactile discrimination in two ROIs (fronto-central and posterolateral areas). The control group shows a significant decrease in connectivity in both ROIs after training that predominantly affected fronto-central connections. Changes in the deaf participants, in contrast, were more widespread and less specific.

**FIGURE 3:**
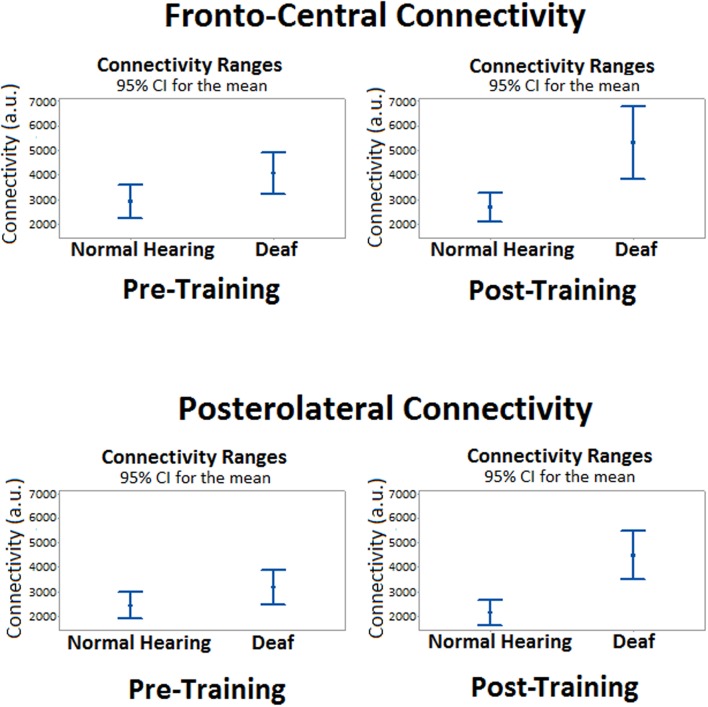
**Mean differences in brain connectivity while processing target stimuli between deaf and NH participants, before and after a short training period in vibrotactile recognition**.

The analysis of the fronto-central region showed significant differences in the mean connectivity values between groups both prior to (*t* = -2.12; *p* < 0.05) and after training (*t* = -3.27; *p* < 0.001). Analysis of the posterolateral region showed no significant between-group differences in the pre-training period, but evaluation of the post-training period did reveal differences in connectivity between groups (*t* = -4.14; *p* < 0.0001). **Figure 3** shows the analysis of connectivity values in the fronto-central and posterolateral regions studied. This analysis suggests that, in general, brain connectivity decreased in the NH group after training, while an opposite trend is observed in the profoundly deaf group, especially in the fronto-central regions. The increase in brain connectivity in the deaf participants in these anterior areas seems to account for the significant differences found between the groups.

An interesting finding was that arbitrary connectivity units estimated for intra-hemispheric relationships were substantially greater than those estimated at inter-hemispheric connections.

**FIGURE 4:**
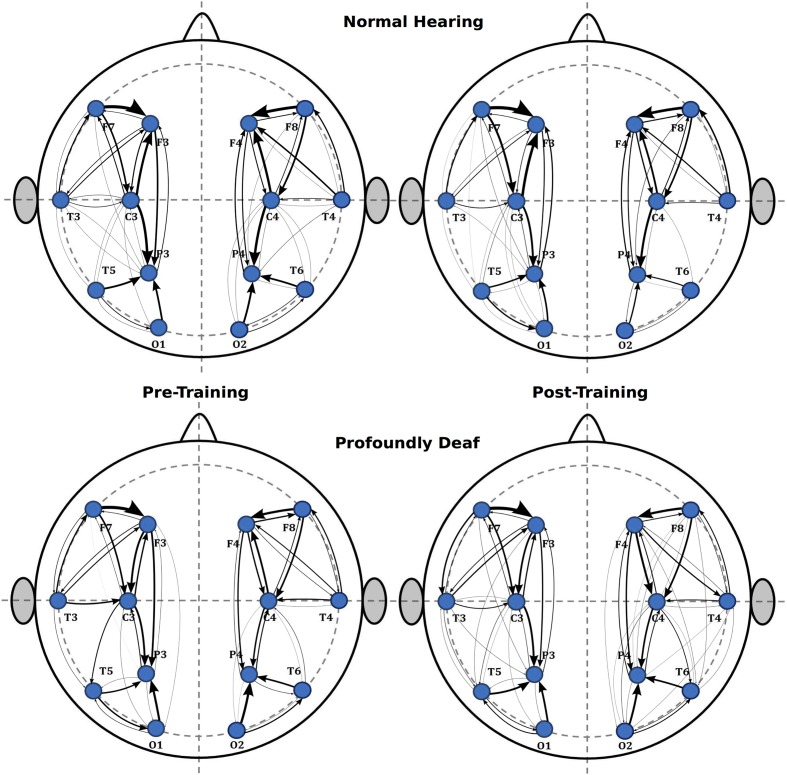
**Sequence of graphs representing brain organization in pre- and post-training conditions in both groups (normal-hearing, profoundly deaf)**. Two brain neural arrangements were studied as main regions of interest: the left and right hemispheres. The graph structure is defined by the probability of connections between brain regions where the thickness of the lines represents the level of connectivity (CPSD × PDC).

Additionally, a tendency suggesting that training might induce greater connectivity in the right hemisphere was observed in the intra-hemispheric analysis, though it did not reach statistical significance in the pre-training period (*t* = 0.62; *p* > 0.05) or after training (*t* = 2.04; *p* > 0.05). As for the left hemisphere connectivity analysis, values showed no significant differences between groups either before (*t* = 1.89; *p* > 0.05) or after training (*t* = 1.53; *p* > 0.05). Finally, **Figure 4** illustrates the main changes in brain connectivity associated with training in vibrotactile discrimination in these two ROIs (left and right hemispheres).

## Discussion

As was expected, both the NH and deaf groups showed similar behavioral performance after a short training period. This is not surprising due to the unimpaired somatosensory capacity of deaf individuals, and the simplicity of the task, despite the novelty of the specific vibrotactile discrimination demands. The widespread activity observed in the auditory cortical regions of the deaf individuals while processing vibrotactile stimuli supports this notion (Auer et al., 2007).

The oddball experimental design used made it possible to interpret the ERP results using the classic P3 framework. Our results indicate a significant increase of the P300 amplitude due to training, even though the between-group amplitude effects did not reach statistical significance, likely due to individual variability. Waveform tendencies show that the voltage magnitude of the P300-like component was slightly higher in the group with profound deafness, even before the training sessions, but increased greatly after training. In both pre- and post-training conditions, the P3 component is seen to be topographically more extended in the deaf group than in NH.

In the oddball paradigm, the P300 component has been interpreted as the result of an orienting response with attention allocation triggered by novel or unusual stimuli that give rise to an “updating” process of stimulus representation (see Polich, 2007 for an extensive review). It has been shown that the amplitude of this ERP component is sensitive to numerous variables and conditions, particularly the amount of attention resources demanded during task performance (Isreal et al., 1980; Kramer et al., 1985), memory engagement (Hartikainen and Knight, 2003; Azizian and Polich, 2007), stimulus modality (Singhal and Fowler, 2005), rare-frequency ratio, task difficulty, mental workload, processing capacity, and such motivational aspects as task relevance and stimulus meaning (Polich and Kok, 1995; Kok, 1997, 2001; Baykara et al., 2016). In this regard, we believe that motivation and relevance were two important variables that influenced neural processing in the deaf group in our study, since learning to discriminate sound through an alternative sensory pathway could have practical applications in their daily lives, whereas in the NH controls this is not the case.

The effects of practice and repetition are key aspects of learning and automatization (Shiffrin and Schneider, 1977; Shiffrin and Schneider, 1984). Braun et al. (2002) proved that repetitive exposure to relevant tactile stimulation on hands and fingers modifies somatosensory neurons, such that the results of behavioral training persist beyond the training period. Electrophysiological changes related to different kinds of training have been described in several studies. Kramer et al. (1986) reported significant component amplitude decreases after several training sessions on a visual search task. However, contradictory findings regarding changes in amplitude have also been discussed in terms of amplitude increases due to training. For example, some studies demonstrated that pre-attentive cortical-evoked potentials reflect training-induced changes; specifically, significant voltage increases in N1 and P2 (Tremblay et al., 2001; Tremblay and Kraus, 2002). These findings are more in line with our results, which are related to a P300 amplitude increase observed after five sessions of vibrotactile discrimination training in both groups.

The graph analysis showed that training-related differences in brain connectivity between the groups were mainly restricted to the frontal neural networks. In an fMRI experiment, Yang et al. (2014) showed that tactile priming engages repetition suppression mechanisms during tactile angle matching, and this process decreased the activation of the fronto-parietal circuit.

The present results confirm our hypothesis that electrophysiological brain dynamic organization differs between profoundly deaf and normal-hearing young adults. Moreover, results from the brief training in vibrotactile discrimination of sound strongly suggest not only the probable recruitment of the primary auditory cortex, as several studies have proposed (Levänen et al., 1998; Auer et al., 2007; Karns et al., 2012), but also a distinct functional brain activation engagement to meet task demands. In this context, previous results have indicated that greater activation of the prefrontal regions and reduced activation of the left parietal cortex might be interpreted as reflecting relatively greater demands on memory and attention resources (Rivera et al., 2005). Therefore, the increase in connectivity observed in the deaf group might reflect the need for greater functional adjustment as a result of training.

Regarding the theoretical view which assumes that incoming stimuli elicit top-down attention switching, while bottom-up memory-drive processes settle on the final outcome (Escera et al., 1998; Goldstein et al., 2002), our results probably depict that profoundly deaf individuals need additional attentional resources to maintain memory items due to task demands. In terms of cognitive resources, the training-induced topographical changes in P300 suggest that NH participants seem to use the available resources more efficiently. This notion coincides with previous reports on visual perceptual skills, which suggest that deaf individuals may allocate their visual resources over a wider range than NH individuals (Sladen et al., 2005). However, they have difficulty in controlling the reallocated visual attention resources (Dye and Hauser, 2014) and they also have a reduced multisensory interaction (Hauthal et al., 2015).

Furthermore, the intra-hemispheric tendency observed in this study showed higher P300 right hemisphere amplitudes in the deaf group. In this context, the lateralization to the right of P300 in those participants might be interpreted as part of a supplementary activation of spatial precision processing mechanisms in personal space, directly linked to the right posterior parietal cortex (Longo et al., 2012), as has been related reiteratively to spatial re-orienting (Roy et al., 2015). The right intra-hemispheric connectivity determined in association with training might underlie the lateralized changes observed in the ERP waveforms. The notion of functional topographic differences between groups might be supported as well by the finding that the right hemisphere has been seen to be more dominant in spatial (tactile pattern) than temporal (vibrotactile frequency) processing in NH individuals (Hegner et al., 2010), though we observed a strong right lateralization in vibrotactile frequency processing in our deaf participants.

The connectivity units estimated for intra-hemispheric relationships were greater than those obtained at inter-hemispheric connections. This could be interpreted as reflecting the effect of attentional modulation on both the primary and secondary somatosensory cortices (Goltz et al., 2013) and, possibly, the construction of mental representations of the target stimuli in order to solve the task; a process in which the right somatosensory cortex seems to play an important role (Schmidt et al., 2014).

Up to now, biased connectivity and shape sensitivity seem to explain plasticity in sensory deprivations (see Heimler et al., 2015, for a comprehensive review). In light of our results, neural relationships following auditory sensory deprivation should be taken into account when studying the potential cross-modal activation of the primary auditory cortex. These relationships might include the activation of novel complex neural ensembles, as was demonstrated recently in animal models (Chabot et al., 2015).

The findings from the present experiment suggest that new emergent attention demands might trigger a task-driven connectivity arrangement in which neural networks comprising frontal, somatosensory and parietal areas could participate. The recent report on the dynamic association between intersensory attention and temporal predictability –as occurred by design in our work– in relation to the shaping of oscillatory power and brain connectivity to facilitate stimulus-processing (Keil et al., 2016) seems to support our explanation. However, we must consider that the broad age range in our sample produced within-group, age-related electrophysiological differences. Indeed, neurodevelopmental ERPs and differences in brain connectivity elicited by early auditory deprivation are interesting phenomena that require future exploration, specifically in younger profoundly deaf populations.

In summary, ERP tools and graph analysis successfully illustrated the differences in the electrophysiological responses –pre- vs. post-training– in vibrotactile discrimination in deaf and NH individuals, and highlighted the neural plasticity capacity in the profoundly deaf. They also revealed the need to recruit additional attention and memory resources. The diffuse resource distribution and regional connectivity observed in the profoundly deaf, before and after training, may also represent a window of opportunity for this way of processing sound via index-fingertip somatosensory stimulation. Vibrotactile sensory feedback in speech production therapy has significant clinical implications for early language development in this population. In future studies, a lengthier training period of vibrotactile sound discrimination, perhaps involving language stimuli, could benefit intermodal brain organization and generate more widespread connectivity. Finally, additional studies are required to clarify the brain connectivity dynamic variation associated with the performance of vibrotactile language-related discrimination tasks with higher cognitive demands.

## Author Contributions

AG-G participated in: research design, data analysis, discussion of the results, and manuscript writing. VR-S participated in: data collection, data analysis, discussion of the results, and manuscript writing. FG-V participated in: research design, data analysis, and discussion of the results. HV-P participated in: data analysis, discussion of the results, and manuscript writing. RR-V participated in: data analysis, discussion of the results, and manuscript writing. RASR participated in: data analysis, discussion of the results, and manuscript writing. AEV participated in: data analysis, discussion of the results, and manuscript writing. LC participated in: data collection, data analysis, and discussion of the results.

## Conflict of Interest Statement

The authors declare that the research was conducted in the absence of any commercial or financial relationships that could be construed as a potential conflict of interest.
