# Supplementary material for: Vibrotactile Discrimination Training Affects Brain Connectivity in Profoundly Deaf Individuals
Source: Front Hum Neurosci. 2017 Feb 6;11:28. doi: 10.3389/fnhum.2017.00028 (PMC5292439; doi:10.3389/fnhum.2017.00028)
Supplement: Supplementary file 1 [file Data_Sheet_1.docx]

**Vibrotactile Sound Discrimination Training Program**

Vibrotactile sound discrimination-training sessions were systematically structured and were imparted by the same instructor. They were one hour in length, with a frequency of two to three times a week all within a maximum three-week period. The sessions focused on training vibrotactile discrimination of frequency and duration properties of sound. The discrimination of a total of twelve more complex sounds, such as natural animal and object sounds was trained. Table 1. specifies how the sessions were organized. Even though activities were repeated throughout the 5 sessions, the difficulty level was progressively incremented tailored individual performance.

| Session | Activities | Time (min) |
| --- | --- | --- |
| 1 | Frequency single pure-tone discrimination | 5 |
|  | Frequency sequence discrimination (F1 and F2) | 10 |
|  | Duration single pure-tone discrimination | 5 |
|  | Duration sequence discrimination (D1 and D2) | 10 |
|  | Animal sound discrimination | 15 |
|  | Object sound discrimination | 15 |
| 2 | Frequency single pure-tone discrimination | 5 |
|  | Frequency sequence discrimination (F1 and F2) | 10 |
|  | Duration single pure-tone discrimination | 5 |
|  | Duration sequence discrimination (D1 and D2) | 10 |
|  | Object sound discrimination | 15 |
|  | Animal sound discrimination | 15 |
| 3 | Object sound discrimination | 15 |
|  | Duration sequence discrimination (D3 and D4) | 15 |
|  | Animal sound discrimination | 15 |
|  | Frequency sequence discrimination (F3 and F4) | 15 |
| 4 | Animal sound discrimination (all six animal sounds) | 15 |
|  | Frequency sequence discrimination (F1, F2, F3, and F4) | 15 |
|  | Object sound discrimination (all six object sounds) | 15 |
|  | Duration sequence discrimination (D1, D2, D3, and D4) | 15 |
| 5 | Evaluation |  |
|  | Frequency sequence discrimination (F1, F2, F3, and F4) | 15 |
|  | Animal sound discrimination (all six animal sounds) | 15 |
|  | Duration sequence discrimination (D1, D2, D3, and D4) | 15 |
|  | Object sound discrimination (all six object sounds) | 15 |

Table 1. Timetable for each training session of vibrotactile discrimination of sound.

**Vibrotactile discrimination of pure-tone frequency and duration**

*Materials*

These particular vibrotactile discrimination abilities were trained using visual representations of pure-tone sequences from the TOT (*Test d Ordre temporal*, Universitè de Montreal, 2005), a test designed to evaluate temporal perception of auditory stimuli (pure tones, a trumpet sound and a vowel sound). This evaluation requires the subject to relate acoustic sequences with a corresponding visual representation. A black bar represents a single tone; the frequency (Hz) domain is represented on the y-axis and time or tone duration (ms) is represented on the x-axis (see Figure 1 for examples).


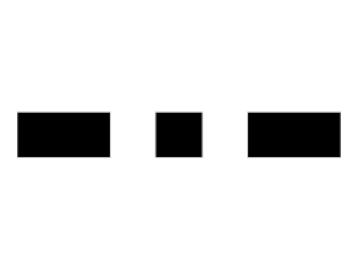

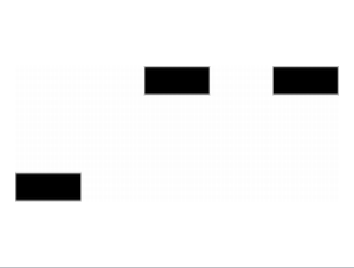


**Figure 1.** Examples of the visual representations of pure-tone sequence combinations from the TOT; each black bar represents a single tone. **Left:** Sequence consisting of 3 pure-tones of the same frequency (1000 Hz) but with different durations (short bars= 250 ms and longer bars= 500ms) and a 300 ms ISI; long-short-long (LSL) sequence combination. **Right:** Sequence consisting of 3 pure-tones of the same duration (200 ms) but of different frequencies (top bars= 1122 Hz and bottom bars= 880 Hz tone) and a 300 ms ISI; low-high-high (LHH) sequence combination.

The TOT has eight different 3-tone sequences for each domain. We adapted these combinations by modifying different variables in the sequence in order to have several levels of difficulty and most importantly to adapt the parameters for vibrotactile perception since the TOT was created to evaluate temporal auditory processing. Using audacity 1.3.3 and Quick Time Player we created 80 different audio recordings, 40 to train tone-duration vibrotactile discrimination and 40 to train frequency discrimination.

The eight 3-tone frequency sequences were: low-high-high (LHH), low-low-high (LLH), low-high-low (LHL), high-low-low (HLL), high-low-high (HLH), high-high-low (HHL), high-high-high (HHH) and low-low-low (LLL). The high frequency tone in this exercise remained constant at 1122 Hz, but the low frequency tone varied: 630 Hz, 730 Hz, 780 Hz, 830 Hz, or 880 Hz. All tones were 200 ms in length, however the inter-stimulus interval (ISI) could be adjusted to 1000 ms, 750 ms or 500 ms. The eight 3-tone duration sequences were: short-short-short (SSS), short-long-short (SLS), long-short-short (LSS), short-long-long (SLL), long-short-long (LSL), long-long-short (LLS), long-long-long (LLL) and short-short-long (SSL). The tone frequency remained constant at 800 Hz and the short tone duration at 250 ms. The long tone could be 650 ms or 500 ms, and the ISI could be adjusted to 1000 ms, 750 ms or 500 ms. Letter-sized flashcards containing 4 sequences were created. There were a total of four different combinations for each domain (frequency: F1, F2, F3, and F4; duration: D1, D2, D3 and D4). The difficulty levels for the frequency discrimination tone sequences are shown in Tables 2 & 3.

| DIFFICULTY | Low-frequency tone (Hz) | Inter-interval stimulus (ms) | | |
| --- | --- | --- | --- | --- |
|  |  | Easy | Medium | Hard |
| LEVEL 1 | 630 | 1000 | 750 | 500 |
| LEVEL 2 | 730 | 1000 | 750 | 500 |
| LEVEL 3 | 780 |  | 750 | 500 |
| LEVEL 4 | 830 |  | 750 | 500 |
| LEVEL 5 | 880 |  | 750 | 500 |

Table 2. Difficulty levels for the frequency discrimination tone sequences.

| DIFFICULTY | Long-tone duration (ms) | Inter-interval stimulus (ms) | | |
| --- | --- | --- | --- | --- |
|  |  | Easy | Medium | Hard |
| LEVEL 1 | 650 | 1000 | 750 | 500 |
| LEVEL 2 | 500 | 1000 | 750 | 500 |

Table 3. Difficulty levels for the duration discrimination tone sequences.

*Procedure*

The general principle for the training was the perception and decoding of the vibrotactile sequences, followed by the pairing of the vibrotactile stimuli to its corresponding visual representation. Participants were trained to discriminate between tones of different frequencies and duration at various difficulty levels and various speeds by repeated exposure to these sequences. At first, the tone sequence was presented vibrotactily 3 times and then the participant was asked to point to the correct option choosing between four sequences visually represented on the flashcard. As this process became more efficient the tone-sequence repetition was no longer necessary. The discrimination difficulty was progressively adjusted throughout the training sessions. An 85% success rate was required to move on to the next level.

**Vibrotactile discrimination of natural complex sounds: animals and objects.**

Audio recordings from real animal and object sounds were selected and edited using Quick Time Player. Six animal (cow, dog, horse, elephant, donkey, lamb) sounds were edited to have a 5-second duration and six object (bell, clock, hammer, ambulance, phone, piano) sounds, a 9-second duration. Individual (9cm x 9cm) color flashcards with the picture of the animal or object were constructed to provide a visual representation. The procedure was very similar to that followed with the tone sequences: rehearsal and repetition pairing vibrotactile stimuli to their visual representation. Animal and object discrimination was trained separately. Participants were first asked to do a relatively simple pairwise comparison, distinguish between a dog barking versus a cow mooing, or a bell ringing versus hammering. Once pair comparisons were done with an 85% success rate then another complex sound stimuli was incorporated until this was done for all six sounds.
