# Supplementary material for: Vibrotactile Discrimination Training Affects Brain Connectivity in Profoundly Deaf Individuals
Source: Front Hum Neurosci. 2017 Feb 6;11:28. doi: 10.3389/fnhum.2017.00028 (PMC5292439; doi:10.3389/fnhum.2017.00028)
Supplement: Supplementary file 2 [file Data_Sheet_2.DOCX]

The interest in studying brain functions through brain connectivity has increased in recent years. Brain connectivity can be classified into anatomical (or structural), functional and effective connectivity. Anatomical connectivity represents structural associations among different morphological elements. Functional connectivity denotes temporal correlations between brain structures. Finally, effective connectivity refers to the estimation of the direct or indirect influence that one neural system exerts over another.

In his work, Sakkalis provides an overview of the most representative measures used to estimate effective and functional connectivity [1]. According to him, the model-based and the data-driven are two techniques used to estimate effective connectivity while in order to study functional connectivity, linear (cross-correlation and coherence) and nonlinear (phase and generalized synchronization) methods and information-based (cross-mutual information and minimum description Length) techniques are computed. In the case of model-based techniques, theoretical models describing interactions between brain areas are proposed based on neurobiological evidence while in data-driven techniques there is not a priori knowledge of spatial or temporal relationships. In order to estimate direct and indirect causal relationships in a multivariate system, two Granger Causality based techniques were developed: Directed Transfer Function (DTF) [2] and Partial Directed Coherence (PDC) [3]. A more detailed discussion about characteristics, limitations and applications of these methods could be found in [1, 4]. Since we are interested in direct causal relationships, in this work the PDC was computed as effective connectivity estimator [5].


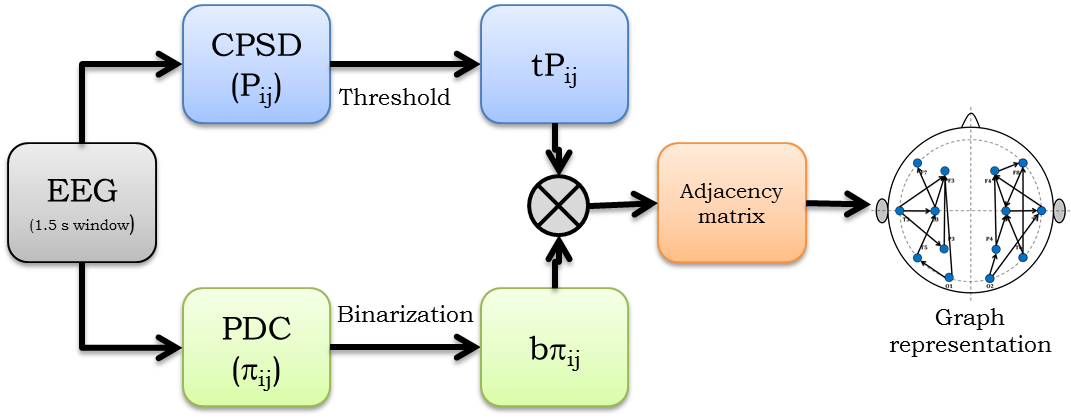


Methodology used to estimate a graph representation.

[1] Sakkalis, V. (2011). Review of advanced techniques for the estimation of brain connectivity measured with EEG/MEG. Computers in biology and medicine, 41(12), 1110-1117.

[2] Kaminski, M. J., & Blinowska, K. J. (1991). A new method of the description of the information flow in the brain structures. Biological cybernetics, 65(3), 203-210.

[3] Baccalá, L. A., & Sameshima, K. (2001). Partial directed coherence: a new concept in neural structure determination. Biological cybernetics, 84(6), 463-474.

[4] Blinowska, K. J. (2011). Review of the methods of determination of directed connectivity from multichannel data. Medical & biological engineering & computing, 49(5), 521-529.

[5] Vélez-Pérez, H., Louis-Dorr, V., Ranta, R., & Dufaut, M. (2008, August). Connectivity estimation of three parametric methods on simulated electroencephalogram signals. In 2008 30th Annual International Conference of the IEEE Engineering in Medicine and Biology Society (pp. 2606-2609). IEEE.
